# Mobile app increases vegetable-based preparations by low-income household cooks: a randomized controlled trial

**DOI:** 10.1017/S1368980018003117

**Published:** 2018-11-26

**Authors:** Peter Clarke, Susan H Evans, Deborah Neffa-Creech

**Affiliations:** Annenberg School for Communication and Journalism, University of Southern California, ASC-324-G, University Park, Los Angeles, CA 90089-0281, USA

**Keywords:** Smartphone app, Fresh vegetables, Low-income, Food pantries, Nutrition

## Abstract

**Objective:**

We built an app to help clients of food pantries. The app offers vegetable-based recipes, food tips and no-cost strategies for making mealtimes healthier and for bargain-conscious grocery shopping, among other themes. Users customize materials to meet their own preferences. The app, available in English and Spanish, has been tested in a randomized field trial.

**Design:**

A randomized controlled trial with repeated measures across 10 weeks.

**Setting:**

Clients of fifteen community food pantry distributions in Los Angeles County, USA.

**Participants:**

Distributions were randomized to control and experimental conditions, and 289 household cooks and one of their 9–14-year-old children were enrolled as participants. Experimental dyads were given a smartphone with our app and a phone use-plan, then trained to use the app. ‘Test vegetables’ were added to the foods that both control and experimental participants received at their pantries.

**Results:**

After 3–4 weeks of additional ‘test vegetables’, cooks at experimental pantries had made 38 % more preparations with these items than control cooks (*P* = 0·03). Ten weeks following baseline, experimental pantries also scored greater gains in using a wider assortment of vegetables than control pantries (*P* = 0·003). Use of the app increased between mid-experiment and final measurement (*P* = 0·0001).

**Conclusions:**

The app appears to encourage household cooks to try new preparation methods and widen their incorporation of vegetables into family diets. Further research is needed to identify specific app features that contributed most to outcomes and to test ways in which to disseminate the app widely.

Vegetables and fruits in the diet contribute to disease prevention^(^^1^^)^. Yet rates of consuming these foods in the USA remain far below recommendations^(^^2^^,^^3^^)^, even in the face of promotional efforts by the federal government costing billions of dollars annually in direct expenditures and contributed resources^(^^4^^,^^5^^)^.

Lack of access to fruits and vegetables has been blamed for low consumption, especially among economically deprived people, although this may have diminished as a barrier^(^^6^^)^. Many low-income US households patronize the more than 33 000 community food pantries nationwide. These pantries channel food to approximately 40 million unique persons per year, food that constitutes more than 4 billion meal-equivalents annually^(^^7^^)^. Fortunately, efforts have succeeded in expanding the supply of fresh produce to clients of food pantries^(^^8^^)^. Escalating prices for fruits and vegetables compared with other foods, however, continue to be vexing for low-income people^(^^9^^,^^10^^)^.

Modest consumption of vegetables may be due to challenges in cutting, seasoning, cooking, storing or combining them with other ingredients^(^^11^^–^^14^^)^. Research suggests that many cooks practise a limited repertoire of preparations, and household members may grow bored and resist repeated servings^(^^15^^)^.

Unfamiliarity with vegetables faced by low-income households inspired the lead authors to create a mobile phone app, *VeggieBook*; the app includes 260 recipes anchored in ten vegetables that are commonly distributed by food banks (J Hager, Manager of Community Health and Nutrition, Feeding America, Chicago, IL, USA, personal communication, 2016). Each recipe was created for ease of preparation, likely availability of ingredients and adaptability when some ingredients are unavailable^(^^16^^)^. The app also features eighty-three vignettes called ‘Secrets to Better Eating’ that provide illustrated, no-cost, evidence-based lessons about healthy food use, happier mealtimes and budget-wise shopping.

The purpose of the present research was to test the effectiveness of our app by determining whether it increased the use of vegetables in preparations of meals and snacks, compared with a control group. The randomized trial ran for approximately 10 weeks in Los Angeles County, USA.

Smartphones seemed a practical means to help shape family diets. Ownership of these devices has trickled down the economic strata; recent data indicate that 64 % of adults in households with annual income less than $US 30 000 own at least one smartphone^(^^17^^)^. Health-related apps have grown in reach and in popularity, too. As one benchmark, by the close of 2014 more than 165 000 apps relating to health or wellness issues were offered on the Google Play Store or Apple App Store^(^^18^^)^. Nearly all are free. Six out of ten smartphone owners have downloaded at least one health app^(^^19^^)^. Studies of health-app use generally show positive correlations with health status, suggesting the possibility of benefits from apps^(^^20^^)^.

Smartphones also offer advantages for communicating information about foods. Being mobile, an app enables just-in-time quests for guidance. Such windows of opportunity arise, for example, when people are deciding what groceries to buy, are coping with unfamiliar vegetables at their food pantry or are starting to fix a meal. Smartphone screens enable brilliant colour displays, important for showing foods^(^^21^^)^ and spurring memory^(^^22^^)^. Apps can be designed to magnify users’ interactive experience, valuable for prompting them to encode and retain information^(^^23^^)^. Apps can present opportunities to exchange content and emotions with others, asynchronously or in real time, adding value to the user’s experience^(^^24^^)^.

Nutrition and cooking apps abound, but in our opinion, most of these seemed ill-suited for low-income users. Often, they present recipes that are too complex or that call for ingredients or equipment that economically strapped families cannot afford. They require elevated skills of literacy or numeracy. They embed kitchen-related help within a thicket of other food or household advice that a cook must plough through to reach information that is immediately action-relevant. Even years after our survey of available apps, a systematic review of diet and nutrition apps found just three that had been empirically tested; these focused on weight loss, not on healthier meal preparations^(^^25^^)^.

Building and testing our app benefited from earlier experience with a tablet-based, message-tailoring system (see point 2 below) that produced printed output for pantry clients^(^^26^^)^. Results from that work convinced us that empowering pantry clients to seek and obtain individually tailored recipe booklets could reap important benefits for frequency of vegetable preparations. Our smartphone app, we hoped, would duplicate the effectiveness of our tablet-based tool while freeing pantry volunteers from having to manage the tablet^(^^27^^)^.

## Materials and methods

### VeggieBook, an app for mobile phones

Our previous formative and evaluation research established design features for the app we built and whose field test is reported here^(^^16^^,^^26^^,^^28^^)^. These included:

1. Launch a user’s quest for recipes by offering a choice among specific vegetables (carrots, green beans, broccoli, etc.). Each pantry client confronts his or her own unexpected supply of vegetables and must cope with that. Users can easily alter their created VeggieBooks as tastes or kitchen skills change. (A VeggieBook is a compilation, or ‘book’, of selected recipes.)
2. Retain the self-profiling questions that our earlier tablet version had successfully used to individualize (tailor) options for recipes and food-use tips. App users encounter five ‘self-profiling’ screens with twenty-one questions at the start of creating each booklet. Screens ask about: (i) preferred cooking methods, in addition to stovetop: microwave, crock pot, juicer, steamer, blender or food processor; (ii) preferred kinds of recipes: kid-friendly, combining the vegetable with a meat, soup, Latino flavoured, Asian flavoured, Soul-Food flavoured; (iii) making snacks, preparing the vegetable for one or two people, making baby food, preparing the vegetable for someone with diabetes; (iv) nutritional tips in general, for children, for adults and seniors; and (v) practical guidance about storing, freezing and preventing spoilage.
3. Add the opportunity to keep or drop recipes that an individual’s self-profiling yielded, providing yet another layer of user choice.
4. Provide colour, paper-based copies of recipes and other content at the pantry, as well as storage on users’ phones. Paper is kitchen-friendly and can be used when other family members have possession of the phone.
5. Enable each user to share recipes with friends, either on paper (printing an extra copy at the pantry) or electronically via email, texting or social media.
6. Create a new, additional set of content, called ‘Secrets to Better Eating’. This section presents eighty-three illustrated, evidence-based ways to improve family meals, shop for food mindfully, involve children in cooking, and more.
7. Invite users to browse websites outside the app, via curated links in ‘Secrets’.
8. Establish a separate access path so that a child in the household can create his or her own VeggieBooks and SecretsBooks to facilitate child–cook conversations about family meals.
9. Enable switching languages (English and Spanish) with a simple toggle function.
10. Build an analytics function that electronically records users’ interactions with the app.

Other details about the app, including samples of screens and printed output, can be found in the online supplementary material, Supplemental File 1. The app can be downloaded free from the Google Play Store (search for ‘VeggieBooks and SecretsBooks’) or from the Apple App Store (search for ‘VeggieSecrets’). A short demonstration can be seen on YouTube (at https://www.youtube.com/watch?v=BBmlMQ2QuEw).

### Study overview and protocol design

The purposes of the present field trial were to: (i) test the app’s effects on the inclusion of vegetables in preparations of meals and snacks, by mothers who patronize food pantries regularly; and (ii) gauge use of the app, where participants were free to use it or not. The agency funding the study, the US Department of Agriculture, required attention to child–mother behaviour surrounding food consumption, so we included a 9–14-year-old in each participating family.

The study design: (i) treated pantry distributions as units of analysis, assigning each programme randomly to control or experimental condition; (ii) accrued as many eligible participants as possible during a typical distribution; (iii) supplemented the pantry foods with standardized bags of fresh vegetables, for the purpose of measuring participants’ immediate use of the vegetables in meals and snacks; (iv) examined use of two ‘target vegetables’ at the end of three weeks, or two at the end of four weeks; (v) gathered baseline and delayed measures of mothers’ use of a wide assortment of vegetables, spanning some ten weeks overall; (vi) provided experimental families with a smartphone (that they kept) with our app and a three-month data plan; (vii) treated experimental and control participants alike in terms of survey interviews and interactions with field staff; and (viii) measured a variety of variables associated with household diets to test whether attrition in research participation across the study’s course undermined comparisons between control and experimental pantry distributions.

The study protocol enrolled fifteen community food pantry distributions in Los Angeles County (out of sixteen invited to collaborate), sites that would permit us to canvass clients to determine their eligibility and willingness to participate. Pantries also had to allow us to supplement the pantry’s foods with our vegetables, distributed just to research participants and not to other clientele, and to occupy scarce space for conducting our client interviews. Pantries mirrored the features of the Los Angeles Regional Food Bank’s roster of 567 affiliated pantries and stretched across all of Los Angeles County. Food distributions varied in size, from serving dozens to hundreds of clients on a typical day; seven were at places of worship and eight were at centres that provide a variety of social services for low-income clients.

We randomly designated six as control distributions and nine as experimental distributions. We wanted to invest the preponderance of study resources where our intervention was likely to create variability in food behaviours.

Eligible participants (mothers or occasionally grandmothers) were regular pantry clients, were their household’s main cook, had at least one 9–14-year-old child living at home who also agreed to participate, had at least one other adult living in the household, spoke and read either English or Spanish, owned at least a basic cell phone (hence excluding families that were inexperienced with mobile technologies), agreed to take part in surveys with their child/grandchild on two weekend occasions at their pantry, and agreed to an additional telephone survey.

**Fig. 1:**
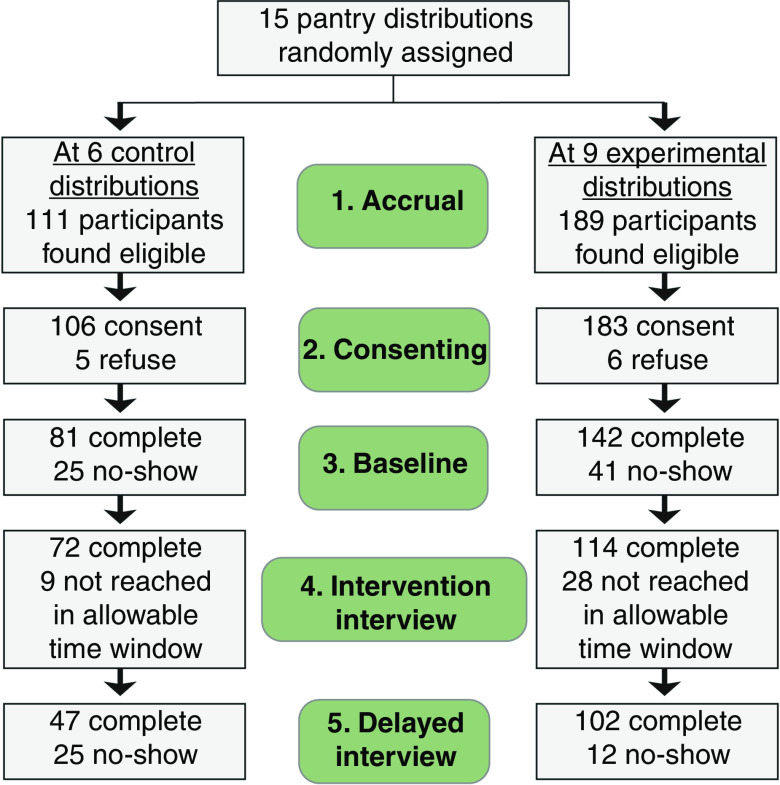
(colour online) CONSORT (Consolidated Standards of Reporting Trials) flow diagram showing the numbers of households retained at five steps of data collection

The CONSORT (Consolidated Standards of Reporting Trials) flow diagram (Fig. 1) shows the numbers of cases at five steps of the study design.

1. Accrual. Potential participants were approached in pantry lines during a regular food distribution to determine eligibility. Attempts to enrol participants continued throughout that day’s distribution.
2. Consenting. On the same day, eligible participants completed a short screening questionnaire and informed consent, harvesting an average of nineteen participants per food distribution.
3. Baseline interview. The mother (or occasionally grandmother, where she was the household’s main cook and source of childcare) and a 9–14-year-old child each participated in face-to-face surveys (conducted by a professional research firm) at the pantry, on a weekend date.
4. Intervention interview. The mother/grandmother participated in a telephone survey (conducted by a professional research firm), six or seven days following either the third or fourth vegetable distribution (see ‘Measurements’ below).
5. Delayed interview. The mother/grandmother and 9–14-year-old child each participated in final face-to-face surveys (conducted by a professional research firm) at the pantry, on a weekend date, five weeks following the end of distributions of supplemental vegetables.

Procedures, questionnaires and paid bilingual field staff were approved and certified by the University of Southern California’s Institutional Review Board.

At consenting, experimental participants were told that they would receive: (i) two different, extra vegetables for each of four weekly pantry distributions; (ii) a Samsung Galaxy S5 smartphone (that they could keep) that was loaded with our app (that would create and store content they selected with the app); (iii) a three-month data usage plan; and (iv) the option to print VeggieBooks and SecretsBooks at the pantry. At consenting, control participants were told that they would receive two different, extra vegetables for each of four weekly pantry distributions thereafter.

At baseline, experimental mothers and their child completed separate, face-to-face interviews and then trained together on the phone and app. It was emphasized that using the app was voluntary, and that participating families would receive their regular pantry foods and our supplemental vegetables regardless of app use. Control mothers and their child also completed separate, face-to-face interviews. Both control and experimental households were given a 5 lb (2·27 kg) bag of potatoes and a bag of red grapes at baseline to accustom them to receiving supplemental provisions from the research project.

Subsequently, control and experimental mothers picked up their weekly, research-supplied allotments of vegetables (along with their usual pantry foods) on a voluntary basis. Across the four weeks of vegetable distributions, in both experimental and control conditions, about one out of ten participants did not attend a distribution. Each week, bilingual project staff asked participants for any food or cooking experiences they wished to share, and staff recorded essentials of conversations that ensued. Clients in the experimental condition could print their VeggieBooks at the pantry during their weekly food pick-ups.

The extra vegetables were: carrots (5 lb) and onions (5 lb) in week 1; sweet potatoes (5 lb) and green cabbage (two heads) in week 2; broccoli (three heads) and green beans (3 lb; 1·36 kg) in week 3; and cauliflower (two heads) and zucchini (5 lb) in week 4.

The vegetables in weeks 3 and 4 are called ‘target vegetables’. These vegetables are the basis for studying the app’s effects on immediate incorporation into meals and snacks. In previous research, these four had been shown to be of medium appeal to low-income cooks^(^^26^^)^.

After week 3, a random half of control and experimental mothers were interviewed by phone about their use of the past week’s vegetables; after week 4, the other half of mothers were interviewed. This division of data collection spread our measure of immediate vegetable-based preparations across four different vegetables. This enhanced the diversity and ecological validity of our assessment of differences in vegetable use.

Finally, all participants were scheduled for a delayed interview at their pantry, five weeks after we stopped giving vegetables and approximately ten weeks after the baseline.

### Measurements

#### Use of vegetables by cooks

We created two distinct ways to measure use of vegetables by mothers: (i) preparations of target vegetables (‘target-veggie preps’ hereafter), at week 3 or 4 of the intervention; and (ii) general vegetable preparations (‘general-veggie preps’ hereafter), measured at baseline and at the delayed interviews.

##### Target-veggie preps

In step 4, respondents were asked in phone interviews whether they recalled receiving and making any use of broccoli or green beans (if contacted after the third supplementary distribution) or receiving and making any use of cauliflower or zucchini (if contacted after the fourth distribution). Surveys were conducted on either day 6 or day 7 following a distribution, to maximize accuracy of recall. Interviewers probed, day by day since the distribution: ‘What did you do with each vegetable on each of the five days? What cooking methods were used for each preparation, if any? Were any fats used, and which ones? Which people in the household had eaten some of the preparations that day, and how much?’

For each participant, we content analysed these combined open-ended and closed-response answers to construct the number of unique preparations that she had made, namely recipes or dishes that were not repeated across the five days. Target-veggie prep scores varied between 0 and 9 preparations, analysing the two vegetables of interest. Reliability of these scores was gauged by recoding the cases, pantry by pantry, blinded to the pantries’ treatment condition. The fifteen reliability scores for the pantries, correlation coefficients between the two separate codings of unique preparations, ranged between 0·86 and 0·94.

We had studied the validity of target-veggie prep scores in earlier, formative studies (SH Evans and P Clarke, unpublished results). One such investigation, for example, compared cooks’ descriptions of preparations that used precise supplies of carrots that we had provided a week earlier against cooks’ counts of carrots that remained in their refrigerators. The correlation was −0·60 (*P* <0·01).

##### General-veggie preps

At steps 3 and 5, in face-to-face interviews, both experimental and control mothers were asked ‘how often in the last seven days’ they had served twenty-seven vegetables at home (from ‘not at all’ to ‘seven or more times’). Mothers were shown colour thumbnail pictures of the vegetables to guard against any unfamiliarity with some names (such as ‘tomatillos’ or ‘jicama’). For purposes of item analysis, a principal components analysis with varimax rotation yielded one dominant factor on which twenty-four of the twenty-seven vegetables loaded with factor loading of >0·30. Vegetables were zucchini, other squash, carrots, lettuce, asparagus, cabbage, cucumbers, green beans, celery, dark leafy greens, broccoli, bell peppers, avocados, root vegetables (like parsnips, turnips or rutabagas), tomatoes, onions, eggplant, potatoes, corn, cactus, jicama, cauliflower, tomatillos and peas. These were summed into a scale (*α* = 0·86). (The three vegetables that did not load were sweet potatoes, chillies and beans other than green beans.)

We consider general-veggie preps as ‘semi-quantitative’ in that it provides ordinal estimates of differences between cooks on vegetable use, low to high, but not necessarily point estimates of the actual frequency of serving any particular item, such as tomatoes or corn. Measurement of general-veggie preps borrows from the vast experiences researchers have gained when developing FFQ assessing consumption^(^^29^^)^. The collection, by self-report surveys, of frequency of consuming specific foods during the past year has been extensively validated against 24 h dietary recalls and other proximate data^(^^30^^,^^31^^)^. It seemed reasonable, therefore, to ask cooks to recall a much shorter time period, the past week, and to tell us how often they had used specific vegetables when preparing meals or snacks.

By assessing general-veggie preps at baseline (step 3) and again at the delayed interview (step 5), we allowed enough lag time for perishable supplies that the study provided to have cleared participants’ refrigerators and cupboards.

#### Self-ratings of VeggieBooks use

As part of phone interviews at step 4, mothers in experimental households were asked ‘to think about VeggieBooks on their phone and in printed booklets’. Specific VeggieBooks (carrots, broccoli, cauliflower, green beans, sweet potatoes and zucchini) were mentioned ‘even though you may not have had a chance to make these yet’. In addition to the four target vegetables, we asked about carrots and sweet potatoes, vegetables that were distributed in earlier studies and that were very popular and very unpopular, respectively^(^^26^^)^. For each booklet that had been made, mothers were then asked ‘how many times’ they had looked at it since: ‘many times, a few times, or not at all?’ Exploratory factor analysis using principal components analysis showed that all six items loaded heavily on one dominant factor (45 % of variance) and items were summed to yield a scale with *α* = 0·76.

#### Self-ratings of SecretsBooks use

During phone interviews at step 4, cooks in the experimental condition were also asked about the five sections (breakfasts, lunches, dinners, snacks, grocery shopping) in ‘Secrets to Better Eating’. Questions mimicked VeggieBooks-use questions (above). Exploratory factor analysis using principal components analysis showed that the five measures loaded heavily on one dominant factor (53 % of variance), and items were summed to yield a scale with *α* = 0·78.

#### Electronic capture of creating VeggieBooks and SecretsBooks

Mothers and their 9–14-year-old child were provided separate Gmail accounts. We tracked the number of times each participant created a ‘booklet’ of content, regardless of whether it was printed at the pantry. These records were tallied across the four weeks of supplemental vegetable distributions at each pantry.

#### Mothers’ talk about food at pantry distributions

When picking up project vegetables, control and experimental participants were greeted by familiar, bilingual (English and Spanish) project staff who asked about ‘any cooking or meal experiences during the previous week that you wish to share?’ These answers were content analysed, blind to treatment condition. Three categories of responses surfaced, mentions of: (i) her household’s eating or enjoyment of any vegetables during the previous week; (ii) having tried new dishes, having expanded her cooking repertoire or having gained new confidence in the kitchen during the previous week; and (iii) her child’s involvement in meal preparations. We coded 285 conversations with control participants, reflecting their 88 % rate of attending food distributions, and 504 conversations with experimental participants, reflecting their 89 % rate of attending food distributions. To gauge coding reliability, we recoded a random 200 of the 789 conversations (approximately one-quarter) and found a 92 % level of agreement.

#### Preferences for printed output or for phone screens

Mothers were asked whether they preferred using printed VeggieBooks or VeggieBooks on their phone screens when ‘planning meals or snacks’, ‘using in the kitchen’ and ‘looking at with their child’.

### Comparability of control and experimental sites

As one would expect, each lag between steps brought some attrition in clients’ participation (see Fig. 1). By step 4, which included measurement of target-veggie preps, a key outcome, 65 % of cases were retained from step 1 at control pantries and 60 % at experimental pantries. Accordingly, we used six aspects of household food use and three demographic variables, all measured at baseline, to gauge the comparability of pantries at step 4. Household measures were: level of household food insecurity; two scales measuring cooks’ level of confidence about kitchen tasks; frequency of serving diverse vegetables for meals and snacks in the past seven days; frequency of serving meal-style convenience foods (e.g. frozen or prepared pizza) in the past seven days; and frequency of serving snack-style convenience items (e.g. crackers) in the past seven days. Demographic variables were mothers’ education, age and size of household (all measures are explained in the online supplementary material, Supplemental File 2).

Means for the two groups of pantries did not differ at step 4 on any of these variables, suggesting that attrition did not undermine experimental comparisons of interest.

### Deviation from original protocol

We started the experiment intending that pantry clients would receive six weeks of study-supplied supplemental vegetables. Three control pantries and one experimental site were fielded using this six-week schedule. Then, it became clear that we lacked budget to sustain the plan. The remaining eleven pantries followed a four-week protocol.

It seems unlikely that combining the four- and six-week protocols distorted experimental differences in favour of the app’s effectiveness. All six control sites were nearly equal on our main outcome measure, target-veggie preps, whether they had experienced four or six weeks of supplemental vegetables. And, as it turned out, the lone six-week experimental site scored next to lowest on target-veggie preps. Change scores on general-veggie preps also did not differ by length of distribution (*P* = 0·73).

### Data analysis

Earlier pilot studies and summative experiments using the tablet-based system and using earlier versions of target-veggie preps as outcomes had yielded effect sizes in the 0·60–0·75 range. To forecast desirable sample sizes for the present field trial, we set *α* (one-sided) at 0·05 and 90 % power, and determined that sixty to seventy cases were required per treatment condition, control and experimental. The protocol exceeded this goal at step 4.

In analyses of app effectiveness, we treated pantry distributions, not individual cooks, as units for statistical comparisons. This is because distributions, not cooks, were randomly assigned to the control or experimental condition. And some cooks within each pantry were randomly assigned to be measured about use of broccoli and green beans (week 3), while others were measured about cauliflower and zucchini (week 4). Accordingly, pantry distributions were comparable.

Other analyses here are based on individual cooks or on children as units of comparison. Statistical tests for core hypotheses about the app’s effectiveness apply non-parametric procedures that do not assume interval scaling or normal distributions. Significance levels for core tests use one-tailed thresholds because we expected that the average outcomes for experimental pantries would exceed average outcomes for control pantries.

## Results

### Effectiveness of the app

**Fig. 2:**
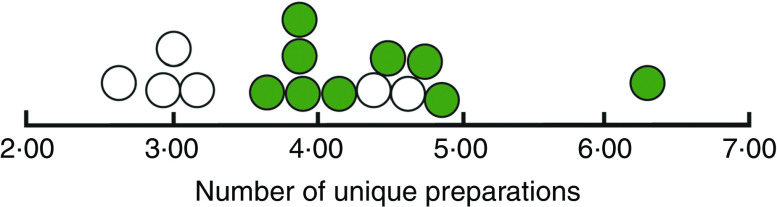
(colour online) Average number of unique preparations, by pantry (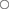, control pantries; 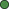, experimental pantries), made by low-income household cooks who were clients of fifteen community food pantry distributions in Los Angeles County, USA, and participated in the randomized controlled trial of the *VeggieBook* mobile phone app with one of their 9–14-year-old children, May 2015–June 2016. Control median = 3·03; experimental median = 4·17 (*P* = 0·03)

Pantry means were computed for target-veggie preps, the number of unique preparations cooks made using broccoli, green beans, cauliflower or zucchini. (Scores within pantries were symmetrically distributed.) Figure 2 shows the average number of preparations for all fifteen pantries, control and experimental. The median of means for six control sites is 3·03, and the median for nine experimental sites is 4·17, 38 % greater. A Mann–Whitney test for differences between pantry ranks was significant at the 0·03 level. Figure 2 also shows that just two control pantries overlapped the distribution for experimental pantries (one had been a four-week site and the other a six-week site; see discussion in ‘Deviation from original protocol’ above). The four other control pantries clustered closely around an average of three preparations using our supplemental vegetables. The Mann–Whitney non-parametric analysis was echoed when we applied generalized linear mixed modelling of the data, considering pantries as fixed effects and households as random effects; this yielded an *F* ratio significant at the 0·004 level, one-tailed.

**Fig. 3:**
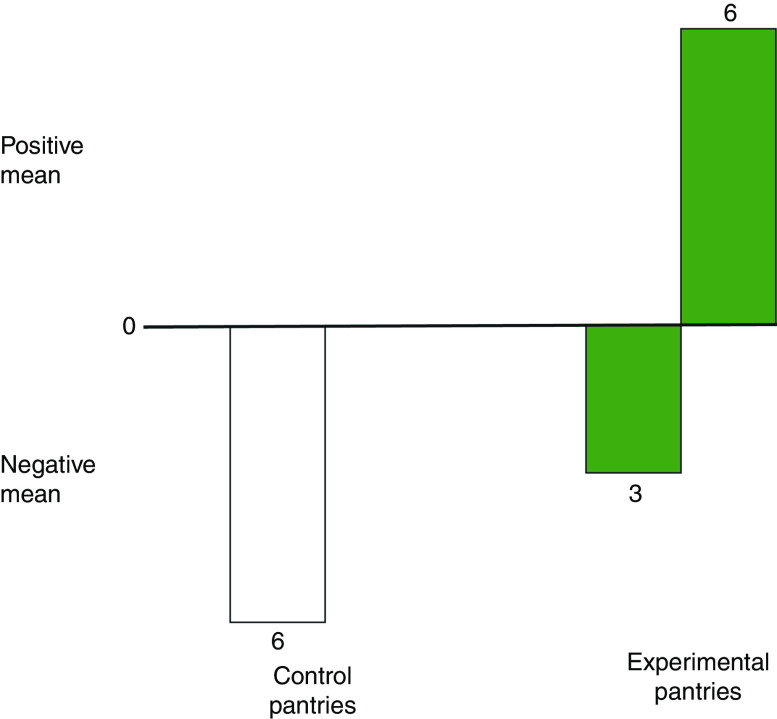
(colour online) Mean difference scores (delayed-interview scores minus baseline scores) for servings of twenty-four vegetables, by pantry, made by low-income household cooks who were clients of fifteen community food pantry distributions in Los Angeles County, USA, and participated in the randomized controlled trial of the *VeggieBook* mobile phone app with one of their 9–14-year-old children, May 2015–June 2016. All six control sites declined in using twenty-four vegetables, on average, whereas six out of nine experimental sites increased their average vegetable usage (*P* = 0·003)

An estimate was also calculated of whether this significant boost in using target vegetables generalized to the use of other vegetables over a longer time span. We compared baseline measures of general-veggie preps (covering twenty-four items) against delayed-interview scores. Simple difference scores, delayed minus baseline, revealed that all six control sites declined in using twenty-four vegetables, on average, whereas six out of nine experimental sites increased their average vegetable usage. Figure 3 shows these results, which are highly significant using Barnard’s test (*P* = 0·003).

An alternative approach tended to confirm this finding. We performed the regression of delayed scores *v*. baseline (*r* = 0·56, *P* <0·01), extracting a residual score for each household cook. Residuals, converted to standard scores, were then averaged for each of the fifteen pantries. Again, we distinguished between positive means, indicating that a pantry increased in vegetable servings more than baseline measures for its clients had predicted, and negative means, indicating that a pantry fell behind in servings, compared with baseline predictions. One out of six control sites had a positive mean, compared with five out of nine experimental sites (*P* = 0·091 by Barnard’s test).

Finally, there were wide differences in changes taking place in control *v*. experimental households, revealed by participants’ weekly conversations with staff distributing the study’s vegetables. Among families with the app, 29 % of staff conversations contained mentions that their families were eating and/or enjoying more vegetables; only 1 % of control conversations said this. In 66 % of experimental conversations, mothers spoke about mastering a greater range of kitchen tasks, or about the more diverse preparations they were able to serve. This compared with 4 % of conversations at control sites. In 39 % of conversations with experimental mothers, they mentioned a child’s involvement in meal preparations: making requests or recommendations for what to cook, helping in the kitchen, setting the table, cleaning up, or participating in other ways. Just 7 % of control conversations made similar mentions about their children (see Table 1).

**Table 1:** At weekly pantry distributions, by pantry, proportions of conversations that included three topics with low-income household cooks who were clients of fifteen community food pantry distributions in Los Angeles County, USA, and participated in the randomized controlled trial of the *VeggieBook* mobile phone app with one of their 9–14-year-old children, May 2015–June 2016

	Control pantries (285 conversations; %)	Experimental pantries (504 conversations; %)
Cook mentions that family is eating/enjoying vegetables more	1	29
Cook says she is gaining confidence in the kitchen	4	66
Child involved in meal preparations	7	39

### Use of the app

**Fig. 4:**
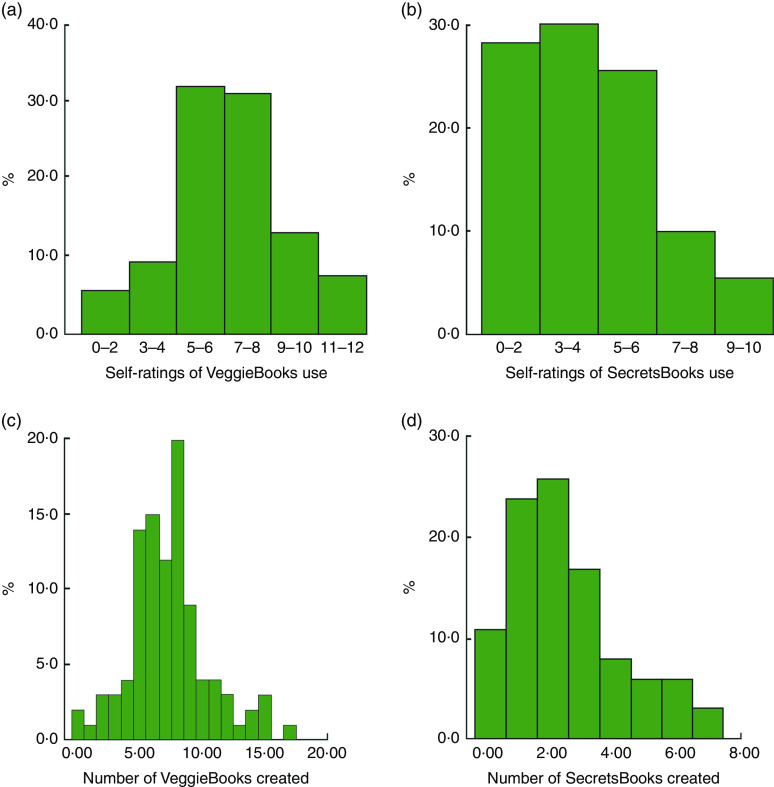
(colour online) Cooks’ use of *VeggieBook* in the experimental pantries: (a) cooks’ self-ratings of VeggieBooks use, scale values (*n* 112); (b) cooks’ self-ratings of SecretsBooks use, scale values (*n* 112); (c) cooks’ number of VeggieBooks created, occasions captured electronically (*n* 110); and (d) cooks’ number of SecretsBooks created, occasions captured electronically (*n* 110). Low-income household cooks were clients of fifteen community food pantry distributions in Los Angeles County, USA, and participated in the randomized controlled trial of the *VeggieBook* mobile phone app with one of their 9–14-year-old children, May 2015–June 2016

Figure 4 shows percentages of cooks arrayed by four measures of app use. The histogram in Fig. 4(a) shows self-reported frequency of making and looking at six VeggieBooks; Fig. 4(b) shows self-reported frequency of making and looking at five SecretsBooks; Fig. 4(c) shows actual occasions of making VeggieBooks, captured by the app’s analytics function; and Fig. 4(d) shows electronically captured occasions of making SecretsBooks.

Cooks’ self-reports about using VeggieBooks were symmetrically distributed and clustered in the mid-zone. Self-reports about using SecretsBooks, on the other hand, were skewed; a handful said that they made no SecretsBooks, but about 70 % made one or more ‘booklets’ and looked at them with varying frequencies. These distributions show cooks’ use of the app during the first three to four weeks of their contact with it. Later, five weeks after supplemental vegetables at their pantries had ended (at step 5 of field research), cooks’ self-reported use of VeggieBooks and of SecretsBooks had increased significantly (distributions not shown here; mean change score for VeggieBooks = 1·6, *P* <0·0001; mean change score for SecretsBooks = 1·7, *P* <0·0001).

Actual counts of making ‘booklets’ are shown in Fig. 4(c) and (d). The modal cook created eight sets of recipes and two types of SecretsBooks, across four weeks.

**Fig. 5:**
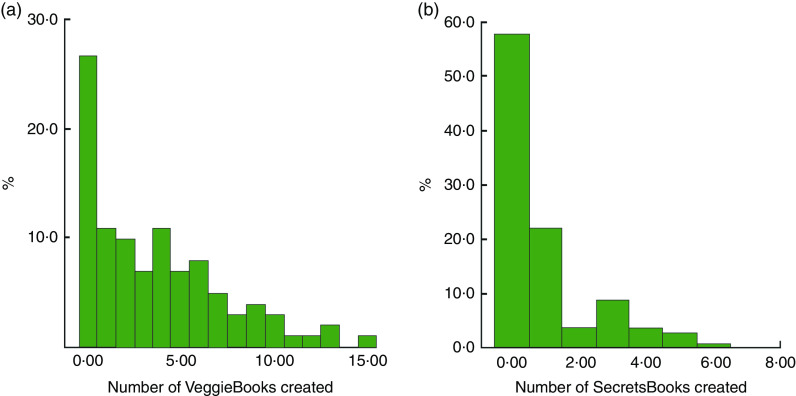
(colour online) Children’s use of *VeggieBook* in the experimental pantries: (a) children’s number of VeggieBooks created, occasions captured electronically (*n* 109); and (b) children’s number of SecretsBooks created, occasions captured electronically (*n* 100). Children aged 9–14 years were from low-income households who were clients of fifteen community food pantry distributions in Los Angeles County, USA, and participated in the randomized controlled trial of the *VeggieBook* mobile phone app with their household cook (mother or grandmother), May 2015–June 2016

Figure 5 shows percentages of children in experimental pantries, arrayed by electronic capture of making VeggieBooks (Fig. 5(a)) and of making SecretsBooks (Fig. 5(b)). On average, children made four booklets of recipes. Nearly half of children made at least one SecretsBook.

Altogether, the typical experimental household, mother and a child, created twelve ‘booklets’ of recipes and three ‘booklets’ of ‘Secrets to Better Eating’. Recipe ‘booklets’ ranged between nine and thirty-six entries, recipes and food tips included, and Secrets ‘booklets’ ranged between eight and twenty-eight vignettes – depending on each user’s preferences at time of creation.

### Preferences for printed output v. phone screens

When planning meals or using VeggieBooks in their kitchens, experimental participants were almost evenly split in their preference for print or phone screen content. About one-third of cooks preferred using VeggieBooks on paper, one-third preferred using their phones and the remainder had no preferences, often volunteering that they liked both versions. For viewing with children, however, mothers expressed a decided preference (62 %) for using output on screen.

### Check on experimental manipulation

The present field trial was artificial in a way that could have contaminated results: experimental participants received a smartphone and a three-month data usage plan. We did this to assure that all experimental families had equal access to the app. We gave phones for them to keep, rather than lending them, so that recipients would be encouraged to incorporate devices into their routine lives.

One might reason that cooks in experimental households began preparing more vegetable-based meals and snacks out of gratitude for our gift. Perhaps the novelty of smartphone ownership, by itself, might have prompted greater use of vegetables.

To check these possibilities, we compared experimental families who already owned a smartphone before enrolling in the study (69 %) with experimental participants who had only a basic cell phone before the study. The average target-veggie prep scores for these two groups did not differ significantly (using individual households as units of analysis, *P* = 0·14). In addition, differences in general-veggie prep scores, delayed minus baseline, were similar between smartphone owners and non-owners (*P* = 0·32). It seems unlikely that the gift of phones and use plans compromised main experimental results.

## Discussion

Results show considerable interest in the app. Mothers/grandmothers, as well as children, became engaged by creating their own VeggieBooks and SecretsBooks. Furthermore, experimental mothers’ self-reports of using both sections of the app increased significantly by the field trial’s end, after they had reverted to a reliance on their pantries’ regular supplies of fresh vegetables. Accelerated use of the app may have bolstered mothers’ growing use of many vegetables, not just ones the experiment distributed. This growth in the app’s apparent value contrasts with the usual finding that health apps wane in interest over time.

Comparisons are difficult to draw, though, between use of our app and use of other nutrition-related apps or recipe databases that are in wide release online. Moreover, evidence about apps in the public domain never reflects use across four weeks by low-income people, the relevant comparison.

A recent and comprehensive mapping of the characteristics of apps for the general public about nutrition and related health^(^^32^^)^ found sixty-four projects to review. Just thirteen of these reports, however, focused on nutrition improvement, actual changes in the quality of food intake. Of these inquiries, none met these two criteria: (i) the app included specific and tailored foods, menus or recipe suggestions to individual users; and (ii) the app was tested for extent of use (or, even better, for effectiveness in achieving behavioural outcomes) among its intended market. Most undertakings in the review were dietary self-monitoring trials, or simply descriptions of apps. Few of the reports included a randomized trial for effects using any kind of quantified outcome of attitudes or behaviour.

Moving beyond raw indices of app use, though, the results from our field experiment seem clear. Data show that having the app and a supply of fresh vegetables, compared with having just a supply of fresh vegetables (control condition), increased cooks’ vegetable preparations decisively, by nearly 40 % in relative terms. But, the app also appears to exert longer-lasting and wider effects, as well. When interviewed five to six weeks after additional, study-supplied vegetables had ended, households in most experimental pantries had increased their use of a broad range of twenty-four vegetables. In contrast, control pantry households did not increase, on average. And, the length of our intervention’s life, approximately ten weeks, lies at the outer boundary of published tests of any nutrition-related app^(^^32^^)^.

Results of our field trial agree with findings from a comprehensive analysis of the effects of phone apps in an array of health domains^(^^33^^)^. Of twenty-three field tests of apps, published in peer-reviewed journals from 2010 to 2015, seventeen projects yielded positive results in such diverse themes as diabetes control, weight reduction, the management of medications and mental health issues. Eight of the apps studied afforded their users tailored messaging, as we did.

The mothers’ self-reports about their families’ use of foods align with behaviour we measured. During the four-week period with supplemental, study-supplied vegetables, our experimental mothers commonly remarked to staff that their families were eating healthier. Experimental cooks also described, in their own words, ways that they were gaining new confidence in the kitchen, trying new ways of preparing vegetables, experimenting with new servings and ‘getting out of the old, cooking rut’. And many experimental families reported that their children had become involved in meal choices and preparations. Control cooks rarely voiced any of these improvements, ideas or changes.

Evidence for the benefits of having the app was gathered using a variety of interview methods (personal and phone), different questionnaire wordings, open-ended and closed-responses, and differing time frames (immediate and delayed).

We recognize, nevertheless, that some of the impact of the app may have hinged on extraneous influences. At least three such factors seem plausible, even though two of them were common to both control and experimental cooks. To reiterate, we provided both experimental and control groups with two supplemental vegetables each week, for four weeks. For experimental mothers, recipes and tips about these vegetables were part of the app’s content. With both conditions, the same staff distributed these vegetables, week after week, and these staff initiated friendly conversations that focused on the previous week’s experiences with food and meals.

The fact, therefore, that the app spurred experimental cooks to quickly make more unique vegetable preparations than control cooks, and to embrace a more diverse portfolio of vegetables, might have depended on: (i) linkages between the app and vegetables we provided; or (ii) linkages to occasions for social exchanges about food; or (iii) both. Put more concretely, perhaps the app ‘works’ mainly when cooks can prepare something right away, shortly after getting the app on their phone. And/or, perhaps the app ‘works’ mainly when cooks feel part of a friendly, weekly conversation at their pantry.

Evidence from our field trial suggests, however, that these potentially facilitating factors were probably short-lived, even if they might have contributed to early use of research-provided vegetables. The delayed measure of household vegetable preparations (step 5) was taken five weeks after supplemental vegetables had ended and five weeks after weekly conversations with staff had stopped. This is enough delay so that any fresh vegetables received from the research project would have been exhausted. And, memories of sociable conversations about food would probably have faded. At this delayed point in time, households provided with the app were using more vegetables than at baseline, whereas control households were not. With this five-week interval, furthermore, most of the vegetables that cooks described using (measured across twenty-four vegetables) were probably a combination of purchased and pantry-provided. Our delayed measure suggests that experimental households with the app were acquiring an enduring interest in and/or capability of using diverse vegetables in meals and snacks.

Additional fieldwork would be needed to test more definitively the importance of these two influences.

A third factor, extraneous to the app’s content, was the invitation that children access the app using their own Gmail accounts. This may have primed an opportunity for intra-family, cross-generational collaboration over food, that might bring many benefits, which have not been documented here.

### Unusual features of research design, as possible limitations on results

Research goals and constraints on field costs required us to innovate when developing the research protocol. We sketch four sets of design decisions and some questions they arouse, leading to possible study limitations.

1. Selecting pantry distributions. We made an effort to choose collaborating pantries that were spread geographically and demographically across sprawling Los Angeles County, our home town food bank’s service area. Budget realities of staff time and travel limited our ambition to include even more sites. The delivery of our supplementary vegetables to each control and experimental site was an even greater constraint. These vegetables (contracted from a food wholesaler) needed to arrive within a 30-min window before the opening of each pantry distribution’s service. This meant sixty timed deliveries that were vulnerable to traffic snarls and demands by the food vendor’s other customers for their deliveries. Would allocating project resources to a different balance between number of food supplements, number of sites, number of data collections and number of participants within sites have yielded firmer tests of the app’s benefits?
2. Standardized foods. Household meal preparations vary by foods’ availability. Pantries’ stocks of vegetables were unpredictable, even hours before distributions. Consequently, for some measurements we needed to establish standard bags of fresh produce given to all control and experimental families. We decided to provide four, weekly vegetable distributions (two vegetables each), conducting phone interviews about vegetable use after the latter two distributions. Should we have chosen a different number of vegetable distributions, either before or during measurements?
3. Learning curve in using phones. We created a teaching module to train experimental mothers and their children how to use the phone and app at baseline interview. Some weeks later, mothers took part in their intervention interview. Did we allow enough time for practice in device use, or should we have allowed for more?
4. Repeated measurements. The field trial extended across approximately ten weeks, with face-to-face interviews at steps 3 and 5, and a phone interview at step 4. Should we have followed up with mothers more often, adding even later waves of data collection, to capture more prolonged use of the app than we did?

### Implications for improving nutrition among low-income households

Our experiment’s findings are promising but leave many important questions unanswered. For example, we cannot say how long households would remain interested in using the app, beyond the weeks of contact documented here. Commonly, use of health apps wanes over time, and other challenges from embracing digital tools for health behaviour change have been eloquently described^(^^34^^)^. Furthermore, improvements in family eating that yield measurable health benefits require longer, sustained changes than this randomized field trial tracked. Also, volume or quantity of diverse vegetable-based preparations, which we measured, should be joined with details about the nutritional quality of preparations. Our study relied on relatively crude descriptions of servings, and not on careful calibration of portion sizes, the composition of dishes or nutrient values.

This randomized controlled trial is also silent about questions concerning the app’s prospects for dissemination. We provided participants with app-loaded phones. At present, we do not know the rates at which pantry clients would download the app to their own phones, if the app were promoted at community sites. Unknown, too, is whether or not self-downloading would be superior, prompting greater app use and magnifying the app’s effectiveness, compared with our method of bundling the app and phone as a gift.

Other circumstances when promoting downloading of the app could powerfully influence people’s subsequent use of the app. For example, how important is having an app-related vegetable in hand? What if the app were promoted by volunteers who were similar to clients in age, ethnicity, social class and other characteristics? What if the app were made available at community clinics, instead of food pantries, or at places of worship?

### Final remarks

The builders of the app are currently experimenting with various dissemination partners – including food banks, statewide entities responsible for SNAP-Ed (Supplemental Nutrition Assistance Program – Education) programmes^(^^35^^)^, school districts, health fairs, Promotoras, and cooking and nutrition classes for kids – on ways to promote sustained and effective household use of the app.

## Supplementary material

For supplementary material accompanying this paper visit https://doi.org/10.1017/S1368980018003117. click here to view supplementary material
