# Supplementary material for: Mobile app increases vegetable-based preparations by low-income household cooks: a randomized controlled trial
Source: Public Health Nutr. 2018 Nov 26;22(4):714–25. doi: 10.1017/S1368980018003117 (PMC6521788; doi:10.1017/S1368980018003117)
Supplement: Supplementary file 1 [file S1368980018003117sup001.docx]

**Supplementary material**

**Supplemental File 1**

**Additional Details about the Appearance and Functionality of the App**

The illustrations in Supplemental Fig. 1a-1e show selected screens from the app and selected paper-based output.


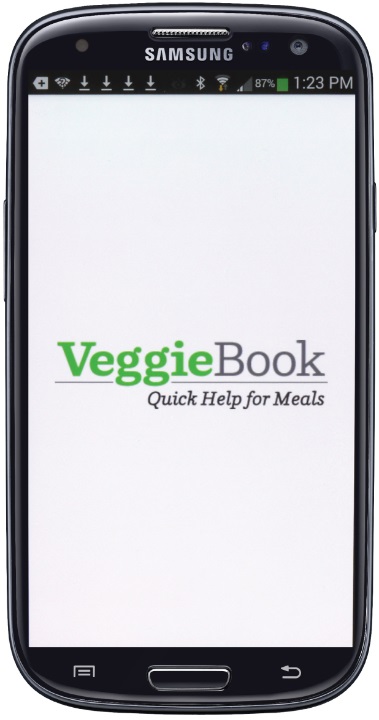

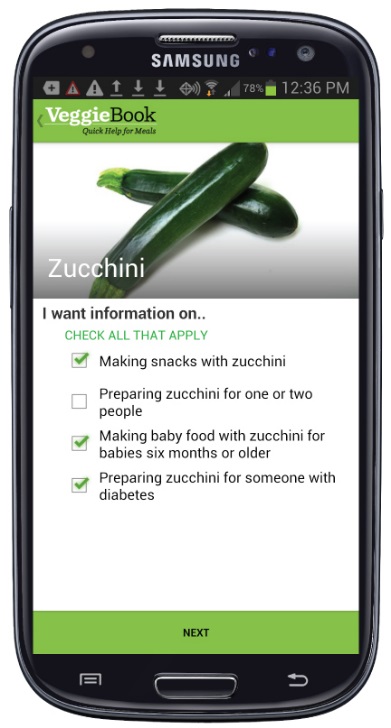


**Supplemental Fig. 1a: Splash page, menu of vegetable options, and one of five self-profiling screens by which a user indicates the kinds of recipes and food tips he or she wants (in this case, in a VeggieBook about zucchini).**


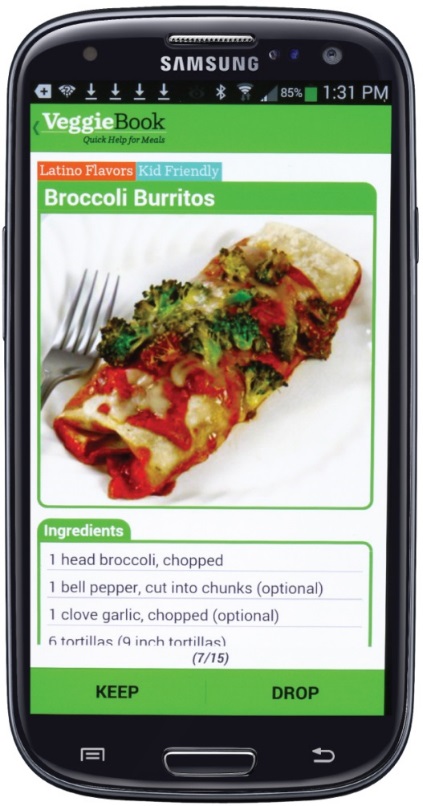

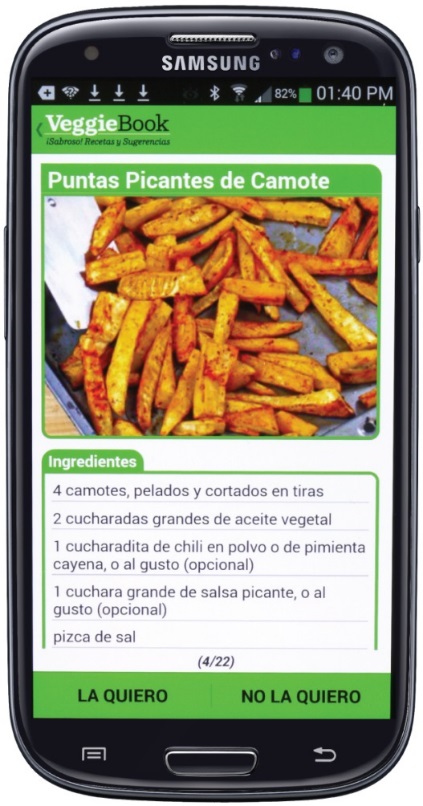

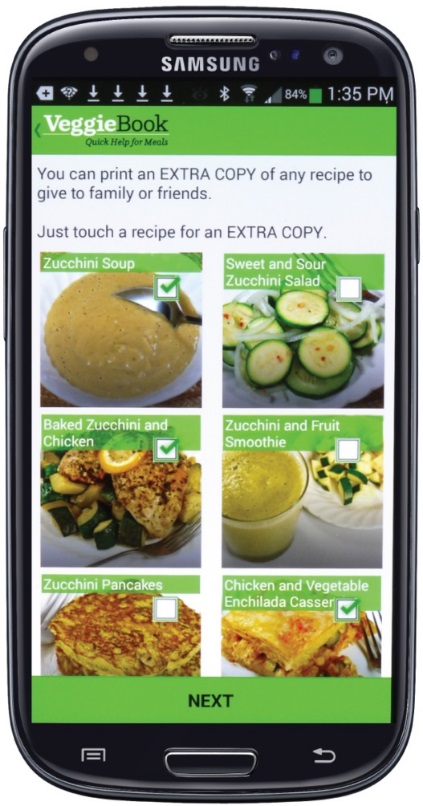


**Supplemental Fig. 1b: A recipe in English, another one in Spanish, and a screen where a user indicates which retained recipes are desired in extra print copies to give friends.**


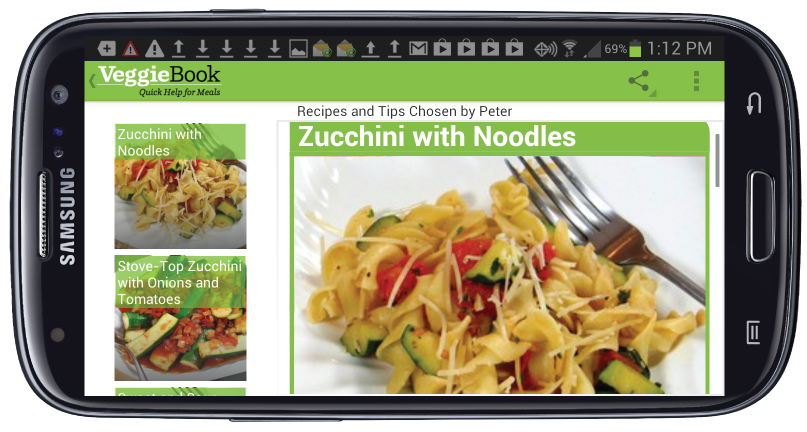


**Supplemental Fig. 1c: The horizontal screen display of a selected recipe**

**along with scrollable thumbnails of all selected recipes**

**Supplemental Fig. 1d: A printed and personalized VeggieBook cover with picture chosen or created by a user, and a printed recipe.**


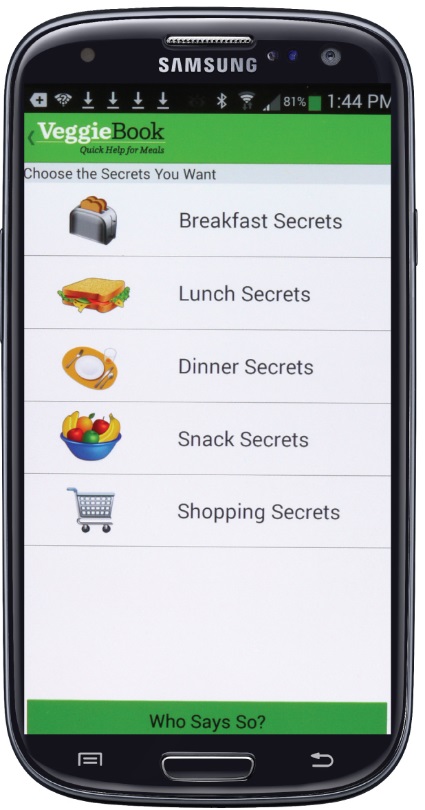

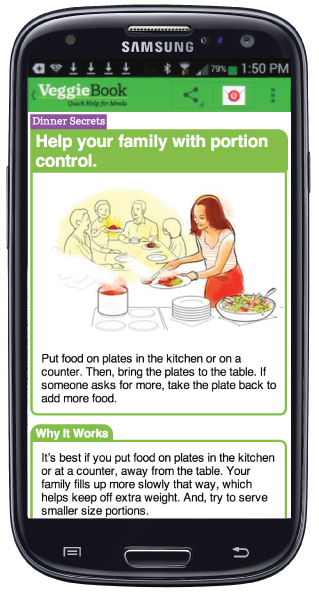

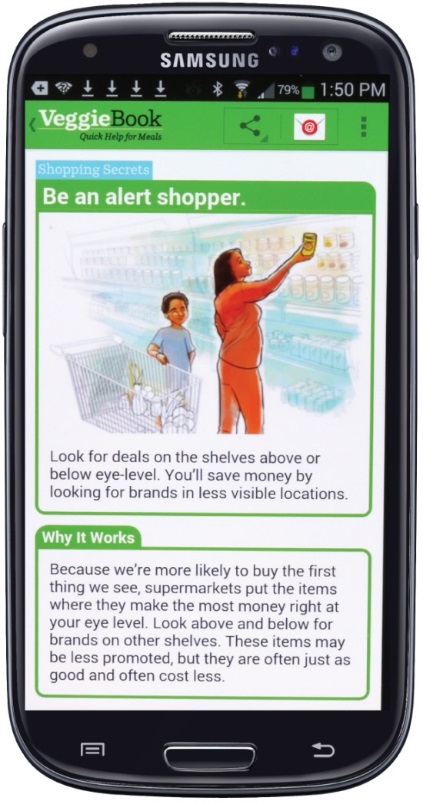


**Supplemental Fig. 1e: Menu for Secrets to Better Eating and two examples of Secrets: One on the importance of plating meals away from the table and the other about searching shelves above and below eye-level when shopping for groceries.**

**Supplemental File 2**

**Variables Used to Test Whether Attrition by Control or Experimental Families Compromised Experimental Comparisons**

Supplemental Table 1 shows mean values for control and experimental pantries and *p*-values from t-tests for differences (two-tailed). Though data were collected at baseline or before (demographic variables), the means are based on participants who were retained at step 4. None of the comparisons is statistically significant. Control and experimental pantries were equivalent on six varying measures of food handling or consumption in the household and on three demographic characteristics.

**Supplemental Table 1**

**Comparability of Control and Experimental Pantries at Step 4**

|  | Pantries’ Means | | *p*-value for diffs. |
| --- | --- | --- | --- |
|  | Control  (N=6) | Experimental  (N=9) |  |
| **Food-related measures** |  |  |  |
| Food insecurity | 1.69 | 1.63 | .74 |
| Cooking confidence-1 | 6.34 | 6.59 | .12 |
| Cooking confidence-2 | 5.09 | 5.14 | .83 |
| General-veggie preps | 51.28 | 44.56 | .21 |
| Convenience servings, meal-style | 3.34 | 4.71 | .10 |
| Convenience servings, snack-style | 10.11 | 12.19 | .18 |
| **Demographic measures** |  |  |  |
| Moms’ education | 2.21 | 2.76 | .11 |
| Moms’ age | 2.89 | 2.77 | .48 |
| Size of household | 5.62 | 5.41 | .46 |

Measures.

**Food insecurity.** Six questions adapted from the USDA’s food insecurity scale were asked of cooks at step 3^(1)^. Answers to three of these items ranged in level of difficulty, determined by percentage of cooks who answered affirmatively. They were: “We could not eat the quality or variety of foods we wanted because of a lack of money” in the past 30 days; “The food that we bought just didn’t last, and we didn’t have money to get more” in the last 30 days; and “In the last 30 days, did you or other adults in your household ever cut the size of your meals or skip meals because there wasn't enough money for food?” Answers to these three formed a Guttman scale with reproducibility = .92 and α = .71. Answers were summed into a scale.

**Cooking confidence.** At step 3, cooks were asked about their confidence in doing different tasks in the kitchen (“very confident,” “somewhat,” or “not at all”). A principal component analysis using varimax rotation revealed two dominant factors with items loading > .44. The first factor was composed of three common kitchen tasks (cooking foods the right amount of time, storing fresh vegetables so they don’t spoil, and cutting foods into right sizes for cooking); α = .55. The second factor was composed of three other kitchen experiences that may have been more novel or infrequent to many (preparing foods that are new to you, following a new recipe, and sharpening a kitchen knife); α = .46. Scales were constructed that represented the two factors--Cooking Confidence-1 and Cooking Confidence-2--and these correlated at .26 (p < .01).

**General-veggie preps.** Recent servings of 24 vegetables, determined by methods described in the main text.

**Convenience servings, meal-style and snack-style.** At step 3, cooks were asked how often in the last seven days they had served 21 processed foods and popular beverages (“not at all,” coded 0, to “seven or more times,” coded 7). Cooks were shown thumbnail pictures of the items. A principal components analysis with varimax rotation yielded two dominant factors with item loadings > .30. The first factor was composed primarily of meal-style convenience foods (seven items: frozen pizza, mac and cheese, hot dogs, other packaged meats, salad dressing, spam, and popcorn); α = .53. The second factor was composed primarily of snack-style convenience foods (nine items: candy, sodas, chips and pretzels, crackers, baked sweets, cookies, fruit punch, sports drinks, and deli meats); α = .73. Scales representing the two factors correlated at .39 (p < .01).

**Demographic measures**. Recruitment interviews at step 2 obtained level of education (1 = primary or less; 2 = grades 7 or 8; 3 = grades 9 - 12; 4 = some college or more); and age (1 = 20s; 2 = 30s; 3 = 40s; 4 = 50s or more). We also took a census of adults and children living in the household.

**References**

1. United States Department of Agriculture (2017) Food security in the U.S. <https://www.ers.usda.gov/topics/food-nutrition-assistance/food-security-in-the-us/measurement.aspx> (accessed March 2018).
